# Developing Reporting Guidelines for Studies of HIV Drug Resistance Prevalence: Protocol for a Mixed Methods Study

**DOI:** 10.2196/35969

**Published:** 2022-05-13

**Authors:** Cristian Garcia, Nadia Rehman, Daeria O Lawson, Pascal Djiadeu, Lawrence Mbuagbaw

**Affiliations:** 1 Department of Health Research Methods, Evidence, and Impact McMaster University Hamilton, ON Canada; 2 Department of Anesthesia McMaster University Hamilton, ON Canada; 3 Department of Pediatrics McMaster University Hamilton, ON Canada; 4 Biostatistics Unit Father Sean O'Sullivan Research Centre St Joseph's Healthcare Hamilton, ON Canada; 5 Division of Epidemiology and Biostatistics Department of Global Health Stellenbosch University Cape Town South Africa

**Keywords:** HIV, drug resistance, reporting guideline, prevalence, surveillance, antiretroviral therapy, report, global health, problem, antiretroviral therapy

## Abstract

**Background:**

HIV drug resistance is a global health problem that limits the effectiveness of antiretroviral therapy. Adequate surveillance of HIV drug resistance is challenged by heterogenous and inadequate data reporting, which compromises the accuracy, interpretation, and usability of prevalence estimates. Previous research has found that the quality of reporting in studies of HIV drug resistance prevalence is low, and thus better guidance is needed to ensure complete and uniform reporting.

**Objective:**

This paper contributes to the process of developing reporting guidelines for prevalence studies of HIV drug resistance by reporting the methodology used in creating a reporting item checklist and generating key insights on items that are important to report.

**Methods:**

We will conduct a sequential explanatory mixed methods study among authors and users of studies of HIV drug resistance. The two-phase design will include a cross-sectional electronic survey (quantitative phase) followed by a focus group discussion (qualitative phase). Survey participants will rate the essentiality of various reporting items. This data will be analyzed using content validity ratios to determine the items that will be retained for focus group discussions. Participants in these discussions will revise the items and any additionally suggested items and settle on a complete reporting item checklist. We will also conduct a thematic analysis of the group discussions to identify emergent themes regarding the agreement process.

**Results:**

As of November 2021, data collection for both phases of the study is complete. In July 2021, 51 participants had provided informed consent and completed the electronic survey. In October 2021, focus group discussions were held. Nine participants in total participated in two virtual focus group discussions. As of May 2022, data are being analyzed.

**Conclusions:**

This study supports the development of a reporting checklist for studies of HIV drug resistance by achieving agreement among experts on what items should be reported in these studies. The results of this work will be refined and elaborated on by a writing committee of HIV drug resistance experts and external reviewers to develop finalized reporting guidelines.

**International Registered Report Identifier (IRRID):**

DERR1-10.2196/35969

## Introduction

### Background

An estimated 38 million people were living with HIV worldwide in 2019 [1]. These large numbers reflect higher longevity in people with HIV due in part to improvements in the management of HIV infection by early detection and early treatment with antiretroviral therapy. One obstacle to the effectiveness of antiretroviral therapy is drug resistance, as it limits the number of effective drugs, increases the potential for onward transmission, and compromises survival [2,3].

Drug resistance to antiretroviral therapy may be acquired when there is viral replication in the presence of a drug [4]. In some individuals, drug-resistant viral strains are already present prior to the start of antiretroviral therapy, referred to as pretreatment drug resistance [5]. This type of resistance can arise due to infection with a drug-resistant viral strain, also referred to as “transmitted drug resistance,” or due to prior exposure to antiretroviral treatment (eg, women and children exposed to treatment as part of prevention programs and people who abandoned prior treatments) [6].

HIV drug resistance is a recognized global health problem [7]. People with drug resistance are more likely to experience treatment failure, discontinue treatment, and develop new drug-resistant strains [5]. The rise in drug resistance is one of the greatest threats to global health—without urgent attention, it could result in millions of deaths, an increase in new harder-to-treat strains of HIV, and higher health care costs [8]. The prevalence of HIV drug resistance varies worldwide, and it can be as high as 25% in some countries [9], likely due to the efforts to expand widespread availability of antiretroviral therapy in these settings. Understanding the levels of HIV drug resistance is important to researchers, clinicians, and policy makers because this information can inform guidelines on how treatment should be tailored and what drugs should be used as first-line treatments. For example, in 2020, a total of 21 of the 30 World Health Organization (WHO) drug resistance surveys reported drug resistance to nevirapine or efavirenz in populations initiating first-line antiretroviral therapy above 10% [10].

The prevalence of drug resistance varies among people living with HIV, but is higher in certain high-risk populations such as men who have sex with men, sex workers, transgender people, people who inject drugs, people in prisons, pregnant women, and adolescents and children; resistance prevalence also varies by sex, ethnicity, and HIV subtype due to differences antiretroviral exposures [11-14]. The pooled prevalence estimate of HIV drug resistance is high among men who have sex with men (13.0%, 95% CI 11.0%-14.0%), sex workers (17.0%, 95% CI 6.0%-32.0%), and people in prisons (18.0%, 95% CI 11.0%-25.0%) [15]. Overall, men who have sex with men are more likely to have any drug resistance compared to the general population (odds ratio 1.28, 95% CI 1.13-1.46) [15].

Adequate monitoring of HIV drug resistance across countries and populations is often challenged by heterogenous and inadequate data reporting. In our previous systematic review of pretreatment drug resistance in key populations, we found that the quality of reporting in studies of HIV drug resistance prevalence is low [16]. This compromises the accuracy, interpretation, and usability of prevalence estimates, especially if key data are not reported, including precision of the estimates, representativeness and diversity of the participants included, techniques used to measure resistance, participants’ transmission risk group, prior exposure to treatments, and class of drug for which resistance was tested [15]. Our recent methodological study concluded that while reporting has improved over time [8], guidance is needed to ensure complete and uniform reporting to improve the interpretation of study findings, generalizability, and comparability of prevalence estimates, while accounting for differences in geographical settings and populations [17].

In 2010, Moher et al [18] published guidance for researchers seeking to develop health research reporting guidelines, outlining a strategy emphasizing the importance of using robust and widely accepted methodologies. In accordance with this strategy and to initiate the process of developing reporting guidelines for studies of HIV drug resistance prevalence, our prior work evaluated the completeness of reporting of HIV drug resistance prevalence literature, the results of which supported the need for reporting guidelines [15,17]. We have registered this guideline project on the EQUATOR (Enhancing the Quality and Transparency of Health Research) network as CEDRIC-HIV (ChEcklist for studies of Drug ResIstanCe in HIV) [19].

### Research Objectives

The objective of this study is to develop a reporting item checklist for prevalence studies of HIV drug resistance by achieving agreement among experts on items that should be reported in studies of HIV drug resistance prevalence. This mixed methods study includes (1) a quantitative phase with survey methodology to identify a list of reporting items considered by participants to be essential, (2) focus group methods to identify emergent themes on reporting items that are essential to HIV drug resistance prevalence studies, and (3) data integration methods to explain discrepancies between quantitative and qualitative data as well as the rationale behind what makes a reporting item important to HIV drug resistance research.

## Methods

### Design

We will conduct a sequential explanatory mixed methods study (QUAN → qual) among authors of studies of HIV drug resistance. This design comprises two phases: a cross-sectional electronic survey (quantitative phase) and subsequent focus group discussions (qualitative phase). The results of the survey will be used to develop an initial list of potential reporting items and additionally suggested reporting items, which will be evaluated, revised, and expanded upon in the qualitative phase. Transcripts from the focus group discussions will provide key agreement-based insights on why these items are important to report.

### Rationale for Design

Mixed methods suit research objectives that cannot met by either qualitative or quantitative methodologies alone [20,21]. The sequential explanatory design is well suited for this research as the quantitative phase provides the recommended reporting items and the qualitative phase provides the rationale for reporting these items. Each of these will inform the guidance and elaboration document that will accompany the checklist.

### Sampling

#### Quantitative Phase

The quantitative phase will include a convenience purposeful sample of corresponding authors of studies of HIV drug resistance. In our 2020 systematic review [16], we searched 10 databases and identified 650 studies of HIV drug resistance. The WHO European region contributed the most studies (34.4%), followed by the Americas (31.7%), Western Pacific (22.0%), and Southeast Asia (6.0%), while African (2.8%) and Eastern Mediterranean regions (1.4%) contributed the fewest studies. We automatically extracted all email addresses (n=160 after deduplication) of the corresponding authors of the included studies. These authors will be contacted by email to participate in the electronic survey. Assuming this is our population of interest, with a 95% CI and a margin of error of 10% and an anticipated survey response proportion of 50%, 84 participants are required. These computations were done with WINPEPI [22]. A sample of n=21 participants will represent ~13% of the target population (N=160), which is sufficiently large to be representative. We intend to recruit as many participants as possible but will use this value to know the minimum required. Study invitations will be sent to all 160 email addresses. If response rates are lower than anticipated, we will use a snowballing approach and invite authors to share the link to the survey with their coauthors. In addition to using social media platforms to disseminate the survey link, HIV journals will also be contacted to share the survey link to authors who have published research on HIV drug resistance in their respective journals.

#### Qualitative Phase

All survey participants will be asked to indicate if they are interested in the focus group discussions. In the qualitative phase, we intend to include a sample of 20 survey respondents who agreed to participate in the focus group discussion (2 groups of 10 participants). We will select these participants with considerations of sex and geographical diversity, such that we have at least one male and one female participant from as many of the 6 WHO regions as possible: African Region, Region of the Americas, South-East Asia Region, European Region, Eastern Mediterranean Region, and Western Pacific Region [23]. We chose to divide participants into 2 groups of 10 to maximize spontaneity and interaction among participants [18]. In addition, research indicates that groups of at least 6 participants are more reliable while groups greater than 12 are logistically more difficult to coordinate [24,25].

### Data Collection

#### Quantitative Phase

Authors of drug resistance prevalence studies will be invited to take an electronic survey on the Research Electronic Data Capture (REDCap) tool hosted at St. Joseph’s Healthcare Hamilton and open from November 2020 to June 2021. REDCap is a secure, web-based application designed for data capture in research [26]. The survey will be pilot tested by the research team prior to launching. Participants will be presented with an overview of the study, its purpose, the investigators, the privacy and confidentiality of their data, and their rights as research participants. They will also be informed on how long the survey will take. Participants will be given the opportunity to provide or refuse consent to participate and the opportunity to withdraw at any time.

The survey includes 23 three-scale ordinal questions, one for each potential reporting item. These 23 items were selected in our previous methodological assessment of reporting completeness of HIV drug resistance prevalence research [17]. This list is not exhaustive, and participants are invited to add more items. Participants will rate whether each item is “essential,” “useful but not essential,” or “not necessary.” Survey items are grouped into four sections in the following order: study-level items, participant items, HIV resistance testing items, and other items. A copy of the electronic survey is provided in Multimedia Appendix 1. This list was generated from a previous systematic review on the global prevalence of HIV in key populations [16]. At the end of each section, participants will be prompted to enter any additional items they believe should be reported, if applicable, into a free-text field. We will also collect basic sociodemographic data such as age, sex, country of residence, profession, number of years as a researcher, and interest in participating in the focus group discussion. Response rates in electronic surveys are often low [27], and thus to maximize responses we will ensure that the email addresses used are up to date, keep the survey as short as possible, declare the estimated time required to complete the survey, and send at least 2 reminder messages [28].

#### Qualitative Phase

Selected individuals who expressed interest in participating in the survey and who consent to being contacted will be approached to set up a convenient time for a group discussion in October 2021. Participants will be given the opportunity to provide consent prior to discussions and for the discussions to be recorded. Interviews will be conducted over Zoom (a videoconferencing platform with real-time messaging and content sharing). The discussions will be moderated by a chair who will ensure that participants are able to contribute freely and openly. The moderator will introduce the session and initiate the discussions based on a focus group discussion guide (see Multimedia Appendix 2). During the discussions, participants will review the initial list of reported items from the quantitative phase and confirm their choice of whether the items are essential. Participants will also review all additionally suggested reporting items brought up in the survey. Although the focus group discussions are not anonymous, participants will be reassured of the confidentiality of their information and that no information provided will be traced back to them. The Zoom sessions will be recorded, with the corresponding recordings/transcripts being stored on secure and password-protected servers. The discussions will last about 2 hours. Agreement will be inferred when at least one participant verbally evaluates whether a reporting item is essential or not and there are no verbal objections with the statement.

### Data Analyses

#### Quantitative Phase

Baseline data and outcomes will be summarized as counts (percentage) for categorical variables, and mean (standard deviation) or median (first quartile, third quartile) for continuous or discrete variables as appropriate depending on the distribution. The ordinal data from potential reporting items will be used to compute a validity ratio. The coding of the essentiality ordinal scale is as follows: essential (3), useful but not essential (2), and not necessary (1). Data on the inclusion of additional reporting items from the open-text fields will be summarized and discussed in the qualitative phase.

A validity ratio will be computed as *VR = [Ne – (N/2)] / (N/2)*, where *Ne* is the number of participants who indicated that the item was essential (ie, a rating of “3”) and *N* is the total number of participants. This ratio will indicate the items that at least half of the participants consider essential. The validity ratio will be interpreted based on a table of critical values [29]. For example, for 20 participants (N=20), the critical value is 0.500 (ie, at least 15 participants must deem the item to be essential). Only items based on a critical value greater than the set threshold will be considered further [30]. This approach facilitates remote and objective decision-making and the estimation of content validity (the degree to which the items represent the construct of complete reporting). We will use the results of the quantitative data to create a draft list of potential reporting items. This list will only contain reporting items with validity ratios above their critical threshold and will be finalized in the focus group discussions.

#### Qualitative Phase

**Figure 1:**
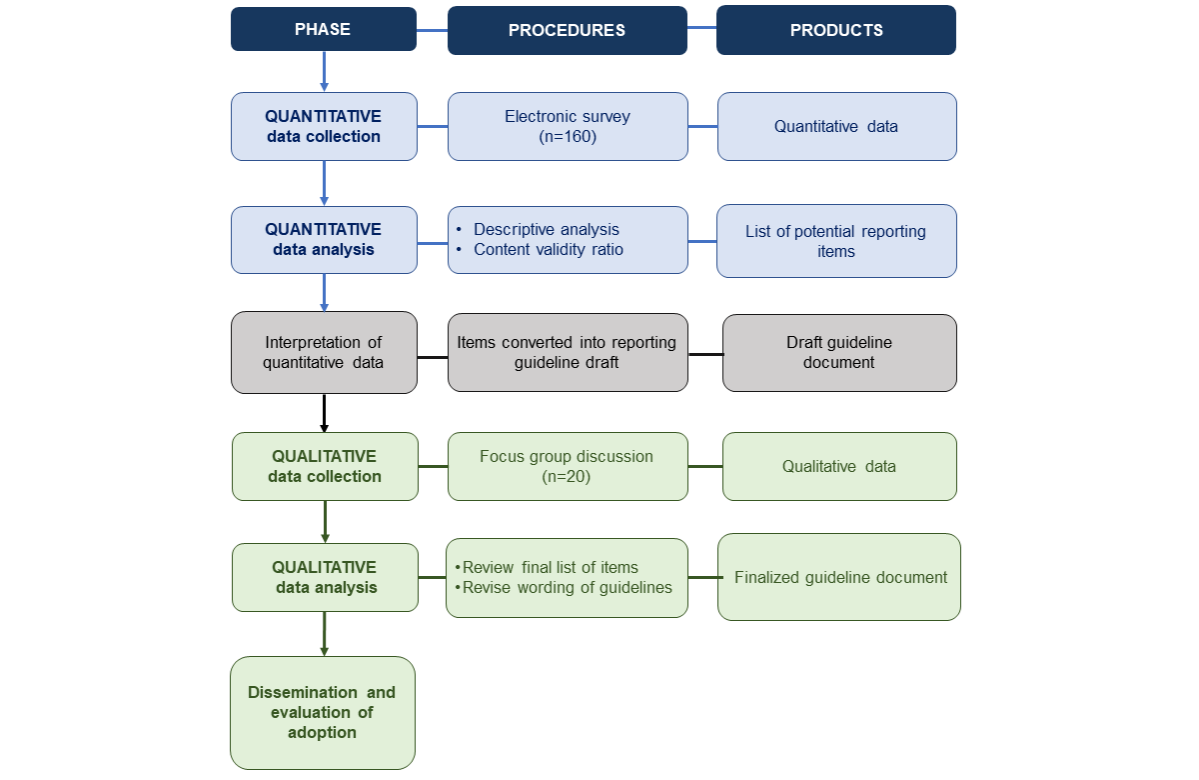
Outline of mixed methods study.

The discussions will be transcribed from recordings and coded into categories by two independent coders and compared for consistency. During the discussions, participants will go over the selected set of reported items and confirm their choice of whether they are essential reporting items. They will also examine the grammar and wording of the items. Participants may propose new items (except items dropped from the survey in the quantitative phase) and these will be discussed. These discussions will be used to finalize the selection of items for the finalized reporting guideline. Qualitative data analysis will be informed by grounded theory, where open codes are generated by identifying repetitions in the text [31]. Similar codes will be grouped, with themes emerging from these groupings. Two coders will verify agreement on the generated themes. Disagreement will be resolved by discussion. Thematic analyses will continue cyclically until no new patterns or themes emerge from the data. An outline of the study is shown in Figure 1.

### Validation Checks

In the quantitative phase, we will pilot test our survey. In the qualitative phase, we will use member-checking, audio-video recordings, and duplicate coding to validate our data. During the focus group discussions, moderator bias will be minimized by using a discussion guide.

### Consensus and Agreement

Consensus will be determined statistically in the quantitative phase using item-specific validity ratios so that the items that at least 50% of participants rated essential are kept in the initial reporting item checklist at the end of the quantitative phase. In the qualitative phase, focus groups will seek agreement on both the reporting item checklist and additionally suggested items generated in the quantitative phase. Agreement is inferred when at least one participant verbally speaks on whether a reporting item was essential or not and there are no verbal objections with the statement. Therefore, agreement also involves the failure to speak up against specific items.

### Ethics Approval

This study received ethics approval from the Hamilton Integrated Research Ethics Board (project number #11558) on November 11, 2020, and received annual renewal approval on September 27, 2021. Only participants who provide informed consent will participate in the study. Participants will be able to stop the electronic survey or withdraw from the focus group discussions at any time.

## Results

**Figure 2:**
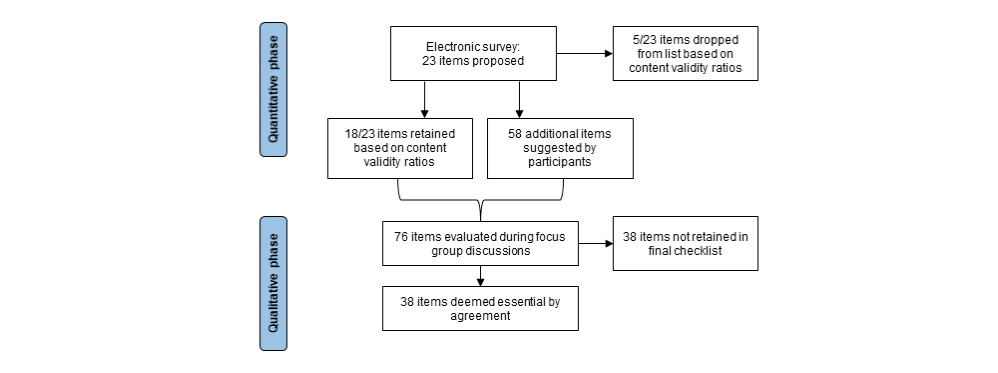
Flow diagram of reporting items dropped and kept in checklist.

The electronic survey was open from November 2020 to June 2021. In total, 51 participants provided informed consent and completed the electronic survey. Once the quantitative phase data collection and analysis was complete, virtual focus group discussions were held in October 2021. We conducted two focus group sessions of 9 participants in total. As of May 2022, results of both the electronic survey and focus group discussions are being analyzed. A flowchart of items dropped and retained in the checklist is provided in Figure 2.

## Discussion

### Overview

In this study, we will use mixed methods to produce a reporting item checklist of items to be considered in the process of developing reporting guidelines for studies of HIV drug resistance prevalence. We will explore and highlight the insights gained from using mixed methods to meet our study objectives. An explanatory sequential design was selected for this study to allow for the use of qualitative data to explain results from the quantitative findings, and breadth and depth in the data collected [32,33].

We anticipate that most of the initially proposed reporting items presented in the survey will be rated as essential and go on to be evaluated in the focus group discussions. We also expect additional reporting items will be suggested by survey participants, which will also be evaluated in the focus group discussions. During the focus group discussions, we expect considerable agreement on the inclusion of most reporting items proposed in the quantitative phase, with disagreements on areas of wording, grammar, and relevance to specific types of HIV drug resistance research designs. As the purpose of this study is to develop a reporting item checklist and key insights to inform the development of reporting guidelines, we anticipate participants will discuss important considerations that the complete reporting guidelines must consider to be accessible and relevant to all authors and users of HIV drug resistance prevalence research, including any concerns over data privacy and confidentiality.

The strengths of this study include the integration of both quantitative and qualitative methodologies to elicit consensus and agreement from experts on the items that should be reported in studies of HIV drug resistance. Additionally, validation checks will be made in both phases of the study to improve data quality. Study limitations include the susceptibility to low response rates in the quantitative phase and therefore the potential for response bias. We have estimated a sample size to determine the minimum number of responses required for the quantitative phase. However, we have specifically incorporated approaches to enhancing diversity of views by reviewing the geographic coverage of the quantitative data, and purposefully selecting participants from high- and low-income settings for the focus group discussions and as external reviewers.

### Dissemination

The results of this work will be presented as peer-reviewed manuscripts, conference presentations, and as part of a master’s thesis. Participants who express interest in the findings of the study will also be sent the results of this work.

### Knowledge Translation

We will incorporate several knowledge translation strategies including engagement of opinion leaders in the agreement discussions (eg, study authors), and through linkage and exchange mechanisms (ie, connecting researchers and knowledge users to facilitate dissemination, for example via educational workshops and project summary briefings to stakeholders) [34]. All focus group participants as well as the individuals who have indicated interest in being informed about the outcomes of this research will be engaged as knowledge user partners to help share the reporting guideline. Additional mechanisms will involve academic media releases (eg, St. Joseph’s Healthcare Hamilton, public health/HIV societies) and web-based social marketing (eg, Twitter). We will also tailor conference meeting presentations to be educational to inform knowledge users (eg, researchers designing HIV drug resistance prevalence studies) about reporting issues and the current gaps at the design stage of HIV drug resistance prevalence studies, and the need for the reporting guideline.

During focus group discussions, we will ask participants about any perceptions of barriers for practice change (eg, at the level of HIV drug resistance prevalence study design) and uptake of the reporting guideline. We will use this feedback to tailor educational activities (eg, conference presentations) and dissemination efforts (eg, preferences for receiving the information) for this audience. For example, to increase awareness about reporting issues and the reporting guideline, we will present findings about the impacts of missing study data, as well as ensure that we target local, national, and international conferences for dissemination activities. We will publish manuscripts arising from this work in open-access journals.

Knowledge translation impact and evaluation will be measured at the level of the HIV research community using the following metrics: reach and use indicators (eg, number of times manuscripts are accessed and cited), collaboration indicators (eg, endorsement by relevant journals in the field), and practice change indicators (eg, improvements in reporting over time) [35]. For example, indicators of uptake will be measured over time in cross-sectional studies to evaluate changes in reporting practices before and after the publication of the reporting guideline.

### Future Directions

The checklist of items and agreement-based insights produced by this study will be refined, elaborated, and considered by a writing committee of experts in HIV drug resistance. We will also invite external reviewers from international organizations such as the WHO, the Joint United Nations Programme on HIV/AIDS (UNAIDS), the Elizabeth Taylor Foundation, and the Centers for Disease Control and Prevention (CDC) to provide feedback on the reporting guidelines.

### Conclusions

We seek to develop a reporting item checklist for studies of HIV drug resistance prevalence and a better understanding of what makes a reporting item important to HIV drug resistance prevalence research. The forthcoming reporting item checklist will directly inform the explanation and elaboration document that will have detailed justifications and rationale for each reporting item in the checklist.

## Appendix

Multimedia Appendix 1 Electronic survey distributed as part of the quantitative phase.

Multimedia Appendix 2 Qualitative interview guide used during focus group discussions.

## Abbreviations

CDC: Centers for Disease Control and Prevention
CEDRIC-HIV: ChEcklist for studies of Drug ResIstanCe in HIV
EQUATOR: Enhancing the Quality and Transparency of Health Research
REDCap: Research Electronic Data Capture
UNAIDS: Joint United Nations Programme on HIV/AIDS
WHO: World Health Organization
