# Supplementary material for: Developing Reporting Guidelines for Studies of HIV Drug Resistance Prevalence: Protocol for a Mixed Methods Study
Source: JMIR Res Protoc. 2022 May 13;11(5):e35969. doi: 10.2196/35969 (PMC9143765; doi:10.2196/35969)
Supplement: Multimedia Appendix 1 [file resprot_v11i5e35969_app1.pdf]

Developing guidelines for studies of pre-treatment HIV drug resistance

Thank you for taking part in our study! This survey should take approximately 12 minutes to complete.

Consent

This survey is administered by Dr. Lawrence Mbuagbaw, Department of Health Research Methods, Evidence and Impact, McMaster University. The purpose of the survey is to develop guidelines for studies of HIV drug resistance by achieving consensus on what items should be reported in studies of HIV drug resistance.

This survey should take approximately 12 minutes to complete. People filling out this survey must be authors of studies on HIV drug resistance.

This survey is part of a study that has been reviewed and approved by the Hamilton Integrated Research Ethics Board (HIREB). The HIREB protocol number associated with this survey is [xx]

You are free to complete this survey or not. If you have any concerns or questions about your rights as a participant or about the way the study is being conducted, please contact:

Office of the Chair, Hamilton Integrated Research Ethics Board at 905.521.2100 x 42013

Having read the above, I understand that by clicking the "Yes" button, I agree to take part in this study	◯ Yes<br>◯ No

About You

What gender do you identify as?	◯ Male<br>◯ Female<br>◯ Other
What is your age?	__________________________

Where is your primary country of residence?

- ◯ Afghanistan
- ◯ Albania
- ◯ Algeria
- ◯ Andorra
- ◯ Angola
- ◯ Antigua & Deps
- ◯ Argentina
- ◯ Armenia
- ◯ Australia
- ◯ Austria
- ◯ Azerbaijan
- ◯ Bahamas
- ◯ Bahrain
- ◯ Bangladesh
- ◯ Barbados
- ◯ Belarus
- ◯ Belgium
- ◯ Belize
- ◯ Benin
- ◯ Bhutan
- ◯ Bolivia
- ◯ Bosnia Herzegovina
- ◯ Botswana
- ◯ Brazil
- ◯ Brunei
- ◯ Bulgaria
- ◯ Burkina
- ◯ Burundi
- ◯ Cambodia
- ◯ Cameroon
- ◯ Canada
- ◯ Cape Verde
- ◯ Central African Rep
- ◯ Chad
- ◯ Chile
- ◯ China
- ◯ Colombia
- ◯ Comoros
- ◯ Congo
- ◯ Congo {Democratic Rep}
- ◯ Costa Rica
- ◯ Croatia
- ◯ Cuba
- ◯ Cyprus
- ◯ Czech Republic
- ◯ Denmark
- ◯ Djibouti
- ◯ Dominica
- ◯ Dominican Republic
- ◯ East Timor
- ◯ Ecuador
- ◯ Egypt
- ◯ El Salvador
- ◯ Equatorial Guinea
- ◯ Eritrea
- ◯ Estonia
- ◯ Ethiopia
- ◯ Fiji
- ◯ Finland
- ◯ France
- ◯ Gabon
- ◯ Gambia
- ◯ Georgia
- ◯ Germany
- ◯ Ghana
- ◯ Greece
- ◯ Grenada
- ◯ Guatemala
- ◯ Guinea

◯ Guinea-Bissau
◯ Guyana
◯ Haiti
◯ Honduras
◯ Hungary
◯ Iceland
◯ India
◯ Indonesia
◯ Iran
◯ Iraq
◯ Ireland {Republic}
◯ Israel
◯ Italy
◯ Ivory Coast
◯ Jamaica
◯ Japan
◯ Jordan
◯ Kazakhstan
◯ Kenya
◯ Kiribati
◯ Korea North
◯ Korea South
◯ Kosovo
◯ Kuwait
◯ Kyrgyzstan
◯ Laos
◯ Latvia
◯ Lebanon
◯ Lesotho
◯ Liberia
◯ Libya
◯ Liechtenstein
◯ Lithuania
◯ Luxembourg
◯ Macedonia
◯ Madagascar
◯ Malawi
◯ Malaysia
◯ Maldives
◯ Mali
◯ Malta
◯ Marshall Islands
◯ Mauritania
◯ Mauritius
◯ Mexico
◯ Micronesia
◯ Moldova
◯ Monaco
◯ Mongolia
◯ Montenegro
◯ Morocco
◯ Mozambique
◯ {Burma}
◯ Namibia
◯ Nauru
◯ Nepal
◯ Netherlands
◯ New Zealand
◯ Nicaragua
◯ Niger
◯ Nigeria
◯ Norway
◯ Oman
◯ Pakistan
◯ Palau
◯ Panama
◯ Papua New Guinea
◯ Paraguay
◯ Peru
◯ Philippines
◯ Poland

	◯ Portugal<br>◯ Qatar<br>◯ Romania<br>◯ Russian Federation<br>◯ Rwanda<br>◯ St Kitts & Nevis<br>◯ St Lucia<br>◯ Saint Vincent & the Grenadines<br>◯ Samoa<br>◯ San Marino<br>◯ Sao Tome & Principe<br>◯ Saudi Arabia<br>◯ Senegal<br>◯ Serbia<br>◯ Seychelles<br>◯ Sierra Leone<br>◯ Singapore<br>◯ Slovakia<br>◯ Slovenia<br>◯ Solomon Islands<br>◯ Somalia<br>◯ South Africa<br>◯ South Sudan<br>◯ Spain<br>◯ Sri Lanka<br>◯ Sudan<br>◯ Suriname<br>◯ Swaziland<br>◯ Sweden<br>◯ Switzerland<br>◯ Syria<br>◯ Taiwan<br>◯ Tajikistan<br>◯ Tanzania<br>◯ Thailand<br>◯ Togo<br>◯ Tonga<br>◯ Trinidad & Tobago<br>◯ Tunisia<br>◯ Turkey<br>◯ Turkmenistan<br>◯ Tuvalu<br>◯ Uganda<br>◯ Ukraine<br>◯ United Arab Emirates<br>◯ United Kingdom<br>◯ United States<br>◯ Uruguay<br>◯ Uzbekistan<br>◯ Vanuatu<br>◯ Vatican City<br>◯ Venezuela<br>◯ Vietnam<br>◯ Yemen<br>◯ Zambia<br>◯ Zimbabwe
What is your primary role?	◯ Research<br>◯ Academia<br>◯ Clinical<br>◯ Industry<br>◯ Government
How long (in years) have you been in this role?	________________________________

Rating Essentiality of Items

The following section will list potential items to be reported in studies of HIV drug resistance prevalence. Please go through each item listed and select a rating of 'essentiality' . Items have been categorized into four categories:
1. Study-level items
2. Participant items
3. HIV resistance testing items
4. Other items

1. Study-level items

	Not necessary	Useful but not essential	Essential
Setting of study (e.g. hospital, community, prison etc.)	○	○	○
Location of study (e.g. country, city, village)	○	○	○
Study design (e.g. cross - sectional, retrospective etc.)	○	○	○
Sample size justification (i.e. was the sample size justified?)	○	○	○

Are there additional study-level items that should be reported?

○ Yes
○ No

Enter these additional study-level items

____

2. Participant items

	Not necessary	Useful but not essential	Essential
Age	◯	◯	◯
Sex/Gender	◯	◯	◯
Sexual orientation	◯	◯	◯
Transmission risk group (e.g. injection drug use)	◯	◯	◯
Profession (e.g. CSW)	◯	◯	◯
Place of residence (e.g. urban, rural)	◯	◯	◯
Ethnicity	◯	◯	◯
Level of education	◯	◯	◯
Income	◯	◯	◯
Exposure to antiretroviral therapy (e.g. treatment-naive)	◯	◯	◯

Are there any additional participant items that should be reported?

◯ Yes
◯ No

Enter these additional participant-level items

____

3. HIV resistance testing items

	Not necessary	Useful but not essential	Essential
Type of resistance test (e.g. Sanger sequencing, next generation sequencing)	◯	◯	◯
Mutation list used (e.g. *WHO SDRM list)	◯	◯	◯
Number of genotypes (as opposed to the number of participants)	◯	◯	◯
Resistance to *NNRTI drug class	◯	◯	◯
Resistance to *NRTI drug class	◯	◯	◯
Resistance to *PI drug class	◯	◯	◯
Resistance to *INSTI drug class	◯	◯	◯
Clinical relevance (e.g. mutations associated with reduced virological response)	◯	◯	◯

*NNRTI: Non-Nucleoside Reverse Transcriptase;
* NRTI: Nucleoside Reverse Transcriptase Inhibitors;
*PI: Protease Inhibitors;
* INSTI: Integrase Strand Transfer Inhibitor;
* World Health Organisation Surveillance Drug Resistance Mutation list;

Are there any additional resistance testing items that should be reported? ◯ Yes ◯ No

Enter these additional resistance testing items

____

4. Other items

	Not necessary	Useful but not essential	Essential
Source of funding	◯	◯	◯

Are there any additional 'other' items that should be reported?

◯ Yes
◯ No

Enter these additional 'other' items

____

Thank you for completing our survey. Better guidance on the reporting of drug resistant HIV will help ensure complete and uniform reporting and improve the appropriate interpretation, generalizability and comparability of prevalence estimates.

Re-contact: Focus Group Discussion

Can we contact you again to participate in a focus-group discussion to finalize a list of items to be reported in studies on the prevalence of HIV drug resistance? This discussion will take place online on Zoom/Skype.	◯ Yes<br>◯ No
Thank you for agreeing to be re-contacted! Please provide your updated contact information based on your preferred methods of contact	
First and last name:	__________________________
Primary email:	__________________________
Secondary email:	__________________________<br>(If applicable)
