# Supplementary material for: Developing Reporting Guidelines for Studies of HIV Drug Resistance Prevalence: Protocol for a Mixed Methods Study
Source: JMIR Res Protoc. 2022 May 13;11(5):e35969. doi: 10.2196/35969 (PMC9143765; doi:10.2196/35969)
Supplement: Multimedia Appendix 2 [file resprot_v11i5e35969_app2.pdf]

McMaster University | HEALTH SCIENCES
Health Research, Methods, Evidence & Impact

Qualitative interview guide

For interviewer:

1. Thank interviewee for participating
2. Introduce yourself
3. Describe the purpose of the interview, benefits and potential harms, permission to record.
4. State the duration of the interview
5. Describe the compensation
6. Determine eligibility
   - Did you take the online survey?
   - Did you express interest in joining the focus group discussion?

If yes to both, proceed to obtain consent and interview.

HIV Expert:

Summary information for focus group:

1. Age
2. Gender
3. Primary role:
   a. Research
   b. Academia
   c. Clinical
   d. Industry
   e. Government
4. WHO region
   a. African Region
   b. Region of the Americas
   c. South-East Asia Region
   d. European Region
   e. Eastern Mediterranean Region
   f. Western Pacific Region

List of potential reporting items and rationale (to be tailored according to quantitative data):

1. Study-level data
   a. Setting of study, e.g. hospital, community, prison etc.
   b. Location of study, e.g. country, city, village
   c. Study design, e.g. cross-sectional, retrospective etc.
   d. Sample size justification, i.e. (was the sample size justified?)
   e. Add any additional items?
   f. Comments on grammar/wording
2. Participant data
   a. Age
   b. Sex/Gender

DRAFT

c. Sexual orientation
d. Transmission risk group, e.g. injections drug use
e. Profession
f. Place of residence, e.g. urban, rural
g. Ethnicity
h. Level of education
i. Income
j. Exposure to antiretroviral therapy, e.g. treatment-naïve
k. Add any additional items?
l. Comments on grammar/wording

3. Information on resistance testing
   a. Type of resistance test, e.g. Sanger sequencing, next generation sequencing
   b. Mutation list used, e.g. *WHO SDRM list
   c. Number of genotypes (as opposed to the number of participants)
   d. Resistance to NNRTI drug class
   e. Resistance to NRTI drug class
   f. Resistance to PI drug class
   g. Resistance to INSTI drug class
   h. Clinical Relevance, e.g. mutations associated with reduced virological response
   i. Add any additional items?
   j. Comments on grammar/wording

   **NNRTI: Non-Nucleoside Reverse Transcriptase; NRTI: Nucleoside Reverse Transcriptase Inhibitors; PI: Protease Inhibitors, INSTI: Integrase Strand Transfer Inhibitor; World Health Organisation Surveillance Drug Resistance Mutation list; *Surveillance Drug Resistance Mutation*

4. Other information
   a. Source of funding
   b. Add any additional items?
   c. Comments on grammar/wording

DRAFT
